# Efficacy and Safety of Platelet-Rich Plasma Injection for Chronic Plantar Fasciitis: A Prospective Study on Functional Restoration and Pain Relief

**DOI:** 10.7759/cureus.52414

**Published:** 2024-01-16

**Authors:** Udit Kothari, Samarth Shah, Deval Pancholi, Chintan Chaudhary

**Affiliations:** 1 Orthopedics, Ashray Orthopaedic Hospital, Modasa, IND; 2 Orthopedics, GMERS (Gujarat Medical Education and Research Society) Medical College, Vadnagar, IND; 3 Orthopedics, Smt. NHL Municipal Medical College, Ahmedabad, IND; 4 Orthopedics, GMERS (Gujarat Medical Education and Research Society) Medical College, Gandhinagar, IND

**Keywords:** chronic plantar fasciitis, vas, aofas, platelet-rich plasma injection, faam

## Abstract

Background

Heel discomfort and functional impairment are frequently caused by plantar fasciitis, and treating it can be extremely difficult for clinicians and occasionally have unfavorable clinical consequences. Recently, platelet-rich plasma (PRP) has been used as an alternative therapy for plantar fasciitis (PF) to reduce heel pain and improve functional restoration. We evaluated the current evidence concerning the efficacy and safety of PRP as a treatment for PF.

Methodology

This was a hospital-based prospective study on patients with plantar fasciitis with a symptom duration of six months or more with failed conservative therapy. All patients included in the study were assessed clinically and by a visual analog score (VAS) for heel pain, the Ankle-Hindfoot Scale (AHS) component of the American Orthopedic Foot and Ankle Society (AOFAS) and Foot and Ankle Ability Measure (FAAM) scores before injection, and at three weeks, three and six-months post-PRP treatment follow-up. Ultrasonography (USG) measurement of plantar fascia thickness was done pre-injection and at the six-month follow-up for clinical outcomes and any complications.

Results

The study included 25 patients with plantar fasciitis, with the majority (48%) in the age group of 21-30 years. Females accounted for 64% of the patients while males accounted for 36%. Most patients (56%) had a moderately active daily activity level. The study found that 16 patients had bilateral plantar fasciitis while nine had unilateral plantar fasciitis. Among the patients with bilateral plantar fasciitis, a total of 32 heels were affected while the 9 patients with unilateral plantar fasciitis had 9 affected heels. Most female patients (75%) had bilateral plantar fasciitis while most male patients (56%) had unilateral plantar fasciitis. Before PRP therapy, both male and female patients reported high pain scores on the VAS for both heels. However, after PRP infiltration, the VAS scores significantly decreased at three weeks, three months, and six months post-injection, indicating pain relief. The AOFAS hindfoot and ankle scores and FAAM scores showed improvement over the follow-up period. Both male and female patients experienced significant improvements in functional outcomes, with increases in AOFAS (p-value 0.45) and FAAM scores (p-value 0.31) at three weeks, three months, and six months post-injection compared to baseline. Statistical analysis revealed a significant decrease in pain scores (73% pain relief), as well as significant improvements in AOFAS scores with an average of 22.33 from baseline (mean = 67.75±9.7) to final follow-up (mean = 90.08±7.9) and FAAM scores with an average of 23.72 from baseline (mean = 49.38±5.2) to final follow-up (mean = 73.10±5.2) after PRP infiltration.

Conclusion

The outcomes of a single dosage of PRP injections demonstrate clinically and statistically substantial improvements in functional outcome scores, plantar fascia thickness evaluated by USG, and VAS scores for heel pain. According to the results of this study, local PRP injection is an effective treatment for chronic plantar fasciitis.

## Introduction

Plantar fascia, which is a thick fibrous aponeurosis originating in the medial calcaneal tuberosity of the heel, as well as the nearby perifascial tissues, undergoes inflammation due to degenerative processes, resulting in plantar fasciitis [1,2]. It is the most common cause of heel pain presenting in the outpatient setting. Although the exact incidence and prevalence of plantar fasciitis by age are unknown, estimations do indicate that the condition accounts for about one million annual patient visits [3]. Around 10% of injuries suffered by runners are caused by this condition, and 11% to 15% of all foot complaints necessitating medical attention are due to it [4,5]. Standing for a long period of time, weight-bearing, or running leads to repetitive strain and tensile overload causing micro-tears of the plantar fascia followed by chronic degeneration due to constant stretching [6,7]. Atrophy of the heel pad, age, obesity, reduced ankle dorsiflexion, tight Achilles tendon, tight intrinsic foot muscles, limb length discrepancy, and excessive foot pronation or flat feet (pes planus) are additional risk factors that play an important role in worsening the condition by increasing the pressure at the plantar surface [8,9].

Due to the complicated regional anatomy and the proximity of probable pain generators, a precise and differentiating diagnosis of plantar fasciitis is usually difficult [10]. There are vascular, infectious, oncologic, and systemic reasons included in the differential diagnosis. The most frequent causes of acute and persistent heel pain are neurologic compression or recurrent microtrauma [11]. Beginning with their initial steps in the morning or after extended periods of rest, patients typically experience start-up pain or plantar medial heel pain. Typically, this pain is acute and does not radiate [12]. Physical examination findings often include soreness with passive dorsiflexion of the first toe and tenderness to palpation on the medial calcaneal tubercle. X-rays or an ultrasound scan can be utilized to diagnose plantar fasciitis if physical examination reveals other injuries or conditions or if the patient doesn't get better after an acceptable period of time [13].

In many aspects, the term "plantar fasciitis" is a misnomer because the disease process is more closely related to a degenerative process like lateral epicondylitis. Histological findings display myxoid degeneration, micro rips, collagen necrosis, and angiofibroblastic hyperplasia rather than any signs of inflammation. These results point to a long-term degenerative process rather than a sudden inflammatory one [10]. In light of this, new therapy regimens that trigger a healing response (PRP) as opposed to stifling an inflammatory process ought to be viable possibilities. Usually, ambulation will improve the heel discomfort, but continuous walking or standing for a long time can worsen the pain by nightfall. Until the symptoms are deemed chronic, patients do not seek medical attention. Although there are several possible treatments for this crippling ailment, none of them have become the accepted course of treatment. As a result, many patients have chronic pain and seek care from several specialists, especially when tried-and-true treatments fail to give the necessary pain relief [14].

Plantar fasciitis is typically treated conservatively, starting with rest and ice to reduce pain and inflammation. Also utilized are non-steroidal anti-inflammatory medicines (NSAIDs), local steroid injections, electrotherapy, and physiotherapy with stretching exercises [15]. Surgical intervention is advised in 10% of instances that do not improve conservative therapy [16].

There is no universally effective treatment for all patients because the cause of plantar fasciitis is still unknown. Most current treatment approaches prioritize reducing inflammation. Rather novel treatment plans that promote a healing response as opposed to stifling the inflammatory process ought to be viewed as more efficient solutions. Platelet-rich plasma (PRP), which is well known to stimulate cell growth and subsequently tissue healing, may help revitalize degenerative tissue by delivering supraphysiologic concentrations of growth factors that catalyze cellular chemotaxis, matrix synthesis, proliferation, and tissue remodeling, and maybe a breakthrough treatment option [17,18].

Due to a lack of sufficient research on the safety and effectiveness of PRP for treating chronic plantar fasciitis in Indian patients, this study aims to assess the effectiveness of PRP infiltration in treating plantar fasciitis in terms of pain reduction, functional outcomes, and its side effects.

## Materials and methods

This prospective study was carried out at the Pt. B.D. Sharma PGIMS at Rohtak's Department of Orthopedics. The study protocol was reviewed and approved by the Institutional Ethics Committee, No. IEC/16/62. The study was carried out following the standards of clinical study as laid down in Schedule Y and the New Drugs and Clinical Trial Act of 2020. This study adhered to ethical guidelines, and potential conflicts of interest were proactively managed. The participants were explained clearly about the nature and purpose of the study in the language they understood and written informed consent was obtained from them. The participants were ensured that their identity would not be revealed at any stage of the study. Regardless of gender, a total of 25 patients who presented to the OPD with plantar fasciitis after a six-week conservative treatment failed were included in the study in which the patients were treated with standard non-invasive treatments including rest, physical therapy, and NSAIDs. Patients with local infection and bleeding dyscrasias, had associated inflammatory enthesitis, such as spondyloarthropathies, cardiovascular, renal or hepatic disease, bacteremia, cellulitis, skin ulceration, vascular insufficiency, malignancy or neuropathy related to the heel, and diabetes mellitus were excluded from the study.

Preparation of PRP

On the day of infiltration, PRP was made at the blood transfusion department using the regular preparation method utilizing the patient's blood. Blood was taken from the patient's anticubital vein a total of 30 ml. After 10 minutes of centrifugation at 3200 rpm, the blood was anti-coagulated in the syringe at a ratio of 1:9 with citrate phosphate dextrose adenine (CPDA).

The RBC layer is discarded and the second centrifuge at 3500 rpm for 10 min yields a more concentrated platelet layer after extraction of platelet-poor plasma (PPP). Two layers of blood were then separated by centrifugation. PPP was in the supernatant while concentrated platelets were in the lower layer. After discarding around three-quarters of the supernatant, the remaining PRP (about 6 ml) was collected into a syringe. The pH was then adjusted by adding 12 cc of sodium bicarbonate. A 21G-1.5" (0.8-38 mm) needle was used to inject the PRP.

Injection technique

An antiseptic solution was used to prepare the area for infiltration, and it was draped sterilely. Prior to administering the regional block, the sites of greatest tenderness were identified. Three to four locations with the most soreness were located by applying pressure with the thumb over the plantar fascia's origin. A skin marker was used to identify these. After injecting two to three cc of local anesthetic, the area was gently massaged with the thumb for 30 seconds. After obtaining a sufficient level of local anesthetic, PRP infiltration was started. A 21 gauge needle was used to pierce each designated area of discomfort until it made contact with the periosteum beneath. As the needle was advanced, a gritty, crunchy quality was audibly and physically felt. The needle was gently partially retracted after making contact with the periosteum, then advanced in a fan-shaped wheel, peppering the area seven to ten times. Continuing this peppering technique, 2mL of PRP was then injected. Afterward, this procedure was completed at each specified location. After infiltration on all the tender places, the operation was finished.

Post-procedure care and follow-up

The infiltration site was covered with a sterile adhesive bandage, and the patient was kept supine for 15 minutes. Ice compression for 20 minutes three times per day and tramadol/paracetamol tablets were recommended for post-procedure discomfort. NSAIDs were stopped for 7 to 10 days, there was no weight bearing permitted for 48 hours, and there was no aggressive running or jumping permitted for 2 weeks. After three weeks, a gradual return to activities was permitted. Follow-up was conducted three weeks, three months, and six months following the procedure, and any type of skin reaction, pain, or complication was observed at this time. During the follow-up period, the outcome was evaluated using the modified AOFAS score, VAS, and FAAM score.

Statistical analysis

The Statistical Package for the Social Sciences (SPSS) software, version 20.0 (IBM Corp, Armonk, NY, USA), was used to analyze the data. The chi-square test (v2) was used to evaluate differences between qualitative variables, and the Mann-Whitney U test and the student's t-test were used to compare differences between quantitative variables in two groups (non-parametric). The analysis of variance (ANOVA) test (parametric) was used to compare multiple quantitative data, and significance was determined by a p-value of 0.05.

## Results

The study was conducted in the Department of Orthopaedics, Pandit Bhagwat Dayal Sharma Post Graduate Institute of Medical Sciences, Rohtak, from August 2015 to October 2017. A total of 25 patients presenting with complaints of plantar heel pain, who did not respond to six weeks of conservative treatment, were included in the study.

Table 1 gives details about the demographic details of the study patients. Out of 25 patients, the majority of the patients (12; 48%) were in the age group of 21-30 years followed by 6 (24%) patients in the age group of 31-40 years of age. Based on gender distribution, 16 (64%) patients were female while 09 (36%) were male in the study. The study found that 14 (56%) of the patients had a moderately active daily activity level, 8 (28%) had a heavy/long-standing daily activity level, and 4 (16%) had a sedentary daily activity level. These findings suggest that the majority of the patients were physically active to some extent with a small percentage sedentary.

**Table 1:** Demographic characteristics of the patients

Age groups (in years)	No. of patients (%)
0-20	1 (4)
21-30	12 (48)
31-40	6 (24)
41-50	3 (12)
51-60	2 (8)
61-70	1 (4)
Gender	
Male	09 (36)
Female	16 (64)
Daily Activity Level	
Sedentary	4 (16)
Moderately Active	14 (56)
Heavy/Long-Standing	8 (28)

Table 2 gives details about the duration of symptoms and the duration of conservative treatment previous to PRP therapy. The mean average duration of symptoms in males was 16.55±7.23 months and 11.97±6.45 months for females. The average mean duration of symptoms in males and females was 13.56±8.38 months. The average duration of conservative treatment previous to PRP therapy in male patients was 8.8±7.23 months and 5.6± 6.45 months for female patients. The average duration of treatment in males and females was 6.7±8.38 months.

**Table 2:** Duration of symptoms and conservative treatment

Duration of symptoms (Months)				
Gender	No.	Minimum(Month)	Maximum(Month)	Mean (Month)
MALE	09	3	36	16.55±7.23
FEMALE	16	4	48	11.97±6.45
TOTAL	25	3	48	13.56±8.38
Duration of treatment previous to PRP therapy (Months)				
MALE	09	2	18	8.8±7.23
FEMALE	16	2	12	5.6±6.45
TOTAL	25	2	18	6.7±8.38

The study examined gender, number, and laterality of affected heels in patients with plantar fasciitis as per Table 3. The results indicate that out of 25 patients, 16 (64%) had bilateral plantar fasciitis while the remaining (9; 34%) had unilateral plantar fasciitis. Among the 16 patients with bilateral plantar fasciitis, a total of 32 heels were affected, while the 9 patients with unilateral plantar fasciitis had a total of 9 affected heels. The majority of female patients (12; 75%) had bilateral plantar fasciitis while most male patients (5; 56%) had unilateral plantar fasciitis.

**Table 3:** Descriptive statistics on the gender, number, and laterality of affected heel in plantar fasciitis

	Total	Unilateral	Bilateral	Affected Heels to Bilateral Patients	Affected Heels to Unilateral Patients	Total Affected Heels
Male	9	5	4	8	5	13
Female	16	4	12	24	4	28
Total	25	9	16	32	9	41

The mean male and female patient’s pre-injection pain scores in the right heel and left heel are shown in Table 4. The VAS score in male patients was 8.50±1.00 for the right heel and 8.89±1.45 for the left heel, while for female patients, it was 9.00±1.00 for the right heel and 8.80±1.14 for the left heel. The AOFAS score in male patients was 69.23±7.05 for the right heel and 69.00±8.20 for the left heel, and for female patients, it was AOFAS scores of 66.33±9.88 and 65.33±11.08 for the right heel and left heel respectively. The FAAM score in male patients was higher compared to female patients for both heels. The study found that for both the right and left heels, there were no significant differences in pain or function scores between male and female patients. However, male patients had slightly higher activity level scores for the left heel. The study also reported that there were no significant differences in the number of affected heels between male and female patients.

**Table 4:** Mean male and female patient’s pre-injection pain score in the right heel and left heel P<0.05 is considered significant. VAS: visual analog score; AOFAS: American Orthopedic Foot and Ankle Society; FAAM: Foot and Ankle Ability Measure

Group Statistics (Right Heel)				
	Sex	No.	Mean Score	P-value
VAS	Male	4	8.50±1.00	0.396
	Female	13	9.00±1.00	
AOFAS	Male	4	69.23±7.05	0.664
	Female	13	66.33±9.88	
FAAM	Male	4	50.25±5.90	0.540
	Female	13	48.46±4.73	
Group statistics (Left Heel)				
VAS	Male	9	8.89±1.45	0.869
	Female	15	8.80±1.14	
AOFAS	Male	9	69.00±8.20	0.400
	Female	15	65.33±11.08	
FAAM	Male	9	51.33±5.38	0.277
	Female	15	48.71±5.56	

The visual analog scores of affected right heels at pre-injection, three weeks, three months, and six months post-injection were 8.88±0.99, 6.59±1.80, 3.53±1.90 and 2.29±1.75, respectively, as shown in Table 5. VAS score decreased after PRP infiltration in plantar fasciitis. The AOFAS hindfoot and ankle score of the right heel at pre-injection, at 3 weeks, 3 months, and 6 months were 69.24±8.95, 73.82±5.72, 85.29±5.59, and 90.94±6.52. The FAAM score of the right heel at pre-injection, 3 weeks, 3 months, and 6 months were 48.88±4.90, 52.12±6.30, 65.82±5.74, and 73.65±4.28. The increase in AOFAS and FAAM scores across the three measurement points showed an improvement in the functional outcome of plantar fasciitis patients.

**Table 5:** Pain relief and functional outcomes (VAS, AOFAS, and FAAM score) in the right heel VAS: visual analog score; AOFAS: American Orthopedic Foot and Ankle Society; FAAM: Foot and Ankle Ability Measure

	Minimum Score	Maximum Score	Mean Score
VAS pre-injection	7	10	8.88±0.99
3 weeks post-injection	2	9	6.59±1.80
3 months post-injection	1	8	3.53±1.90
6 months post-injection	0	6	2.29±1.75
AOFAS pre-injection	51	77	69.24±8.95
3 weeks post-injection	61	85	73.82±5.72
3 months post-injection	71	90	85.29±5.59
6 months post-injection	76	100	90.94±6.52
FAAM pre-injection	42	59	48.88±4.90
3 weeks post-injection	43	68	52.12±6.30
3 months post-injection	54	74	65.82±5.74
6 months post-injection	64	80	73.65±4.28

The visual analog scores of affected left heels at pre-injection, at 3 weeks, at 3 months, and 6 months post-injection were 8.83±1.23, 6.33±1.55, 3.67±1.71, and 2.50±1.79, respectively. VAS scores were lower at all follow-up points than pre-injection scores, showing pain relief reported by patients. AOFAS hind foot and ankle scores of left heel at pre-injection, at 3 weeks, 3 months, and 6 months were 66.71±10.07, 72.04±6.94, 83.58±7.21, and 89.46±8.64. FAAM scores of the left heel at pre-injection, at 3 weeks, 3 months, and 6 months were 49.74±5.53, 53.70±6.51, 65.35±5.89, and 72.70±5.80. The increase in AOFAS and FAAM scores across the three measurement points showed an improvement in the functional outcomes of plantar fasciitis patients (Table 6).

**Table 6:** Pain relief and functional outcomes (VAS, AOFAS, and FAAM score) in the left heel VAS: visual analog score; AOFAS: American Orthopedic Foot and Ankle Society; FAAM: Foot and Ankle Ability Measure

	Minimum Score	Maximum Score	Mean Score
VAS pre-injection	6	10	8.83±1.23
3 weeks post-injection	3	9	6.33±1.55
3 months post-injection	1	6	3.67±1.71
6 months post-injection	0	6	2.50±1.79
AOFAS pre-injection	45	78	66.71±10.07
3 weeks post-injection	48	78	72.04±6.94
3 months post-injection	63	90	83.58±7.21
6 months post-injection	70	100	89.46±8.64
FAAM pre-injection	42	60	49.74±5.53
3 weeks post-injection	42	70	53.70±6.51
3 months post-injection	56	76	65.35±5.89
6 months post-injection	58	80	72.70±5.80

Table 7 shows the mean differences in pain scores between male and female patients. There was no significant difference in VAS pain score between males and females during pre-injection, 3 weeks post-injection, 3 months post-injection, and 6 months post-injection.

**Table 7:** Analysis of the significance of the mean difference in pain scores between male and female patients P<0.05 is considered significant. VAS: visual analog score; AOFAS: American Orthopedic Foot and Ankle Society; FAAM: Foot and Ankle Ability Measure

VAS Score	Sex	Mean	Std. Deviation	P-value
Pre-injection	F	8.89	1.066	0.749
	M	8.77	1.301	
3 weeks post-injection	F	6.79	1.641	0.646
	M	5.69	1.437	
3 months post-injection	F	3.64	1.768	0.863
	M	3.54	1.854	
6 months post-injection	F	2.50	1.795	0.654
	M	2.23	1.739	
AOFAS score				
Pre-injection	F	66.50	10.405	0.233
	M	70.46	7.183	
3 weeks post-injection	F	71.43	7.431	0.348
	M	75.69	1.032	
3 months post-injection	F	83.57	7.244	0.308
	M	85.85	4.688	
6 months post-injection	F	89.39	8.194	0.418
	M	91.54	6.887	
FAAM score				
Pre-injection	F	48.36	5.144	0.138
	M	51.00	5.323	
3 weeks post-injection	F	52.11	6.148	0.106
	M	55.62	6.627	
3 months post-injection	F	64.46	5.433	0.059
	M	68.08	5.664	
6 months post-injection	F	72.14	5.414	0.07
	M	75.23	3.745	

Table 8 shows a regression analysis of results for VAS, AOFAS, and FAAM scores. There are no significant differences in the outcome of VAS, AOFAS, and FAAM scores in any of these three variables based on statistical analysis using a linear mixed-effect model.

**Table 8:** Subgroup regression analysis result for VAS, AOFAS, and FAAM scores (A: symptoms duration ≤ 12 months versus ≥12 months, B: age ≤ 35 years versus ≥ 35 years, C: sex, male versus female) P<0.05 is considered significant. VAS: visual analog score; AOFAS: American Orthopedic Foot and Ankle Society; FAAM: Foot and Ankle Ability Measure

	VAS Score		AOFAS Score		FAAM Score	
Variable	Estimation of coefficient	P-value	Estimation of coefficient	P value	Estimation of coefficient	P value
Duration of symptoms ^A^	1.82	0.892	6.69	0.690	4.32	0.418
Age at injection ^B^	1.75	0.292	6.47	0.270	4.30	0.353
Sex ^C^	1.81	0.799	6.715	0.750	4.42	0.858

For each outcome, pre and post-PRP infiltration, the mean and standard deviation are summarized in Table 9. Following PRP infiltration, the VAS score decreased by an average of 6.44 from baseline (mean = 8.85±1.13) to post-infiltration follow-up (mean = 2.41±1.76), representing a 73% pain relief (p-value = .001). The AOFAS score improved an average of 22.33 from baseline (mean = 67.75±9.7) to final follow-up (mean = 90.08±7.9), a 33% improvement (p-value = .001). Similarly, participants reported clinically significant improvement in FAAM scores, an average of 23.72 from baseline (mean = 49.38±5.2) to final follow-up (mean = 73.10±5.2), a 48% improvement (p-value = .001). No serious complications were reported in our study during PRP infiltration except temporary pain and swelling in 60% of patients for an average of 3 days (range 2-7 days), which subsided gradually within a week.

**Table 9:** Analysis of the difference of the mean pre and post-injection (at the six-month follow-up) pain scores of patients P-value <0.05 as significant. VAS: visual analog score; AOFAS: American Orthopedic Foot and Ankle Society; FAAM: Foot and Ankle Ability Measure

	No	Mean	Std. Deviation	P-value
VAS (Baseline)	41	8.85	1.131	<0.001
VAS (6 Months)	41	2.41	1.760	
AOFAS (Baseline)	41	67.75	9.716	<0.001
AOFAS (6 Months)	41	90.08	7.882	
FAAM (Baseline)	41	49.38	5.222	< .0.001>
FAAM (6 Months)	41	73.10	5.173	

## Discussion

The most frequent cause of heel pain and damage to the plantar fascia is plantar fasciitis [18]. The goal of our study was to evaluate the short-term efficacy and safety of autologous PRP injection in the treatment of chronic PF. Plantar heel pain is caused by a group of elastic and collagenous fibers called plantar fascia, which are linked to the forefoot and originate from the medial region of the calcaneus [19]. Calcaneal spur, a problem that affects many persons of various ages, can be related to heel pain. It is a bony growth on the heel bone with its tip inside the PF origin, which causes the PF to be pulled continuously and causes irritation that results in plantar fasciitis [20].

It is frequently a self-limiting syndrome with symptoms that go away on their own within a year of the commencement, but in other individuals, it progresses chronically and has a major impact on their daily lives and quality of life [21,22]. The genesis and treatment of plantar fasciitis are not entirely known. Pain relief and functional improvement are the major goals of plantar fasciitis treatment. NSAIDs, activity moderation, ice application, arch support, splinting/strapping, deep tissue massage, plantar fascia stretching exercises, and physical therapy are the main conservative treatments for plantar fasciitis [23]. Compared to heat, silicone heel pads, or calf stretching activities, it has been found that plantar fascia stretching exercises significantly reduce symptoms [24].

In 10% of patients, the symptoms continue despite conservative treatment, which results in persistent plantar fasciitis. While they offer positive short-term results, local corticosteroid injections are currently the cornerstone of the management of chronic plantar fasciitis [25,26]. Corticosteroids' anti-inflammatory properties work well to ease heel pain, but they also prevent the growth of fibroblasts and ground substance proteins. Moreover, repeated injections are frequently necessary and are linked to heel fat pad atrophy, plantar fascia rupture or tear, abscesses, osteomyelitis, depigmentation of the skin, and muscle and nerve damage [27,28].

The patient looks for a more contemporary, more successful modality as a result of the current treatment modalities' disappointing results. A novel method for treating plantar fasciitis is a local injection of PRP. Platelet-derived growth factor (PDGF), transforming growth factor beta (TGF-B), insulin-like growth factor (IGF), cytokines, and interleukins are only a few of the growth factors that are abundant in PRP. These growth factors might encourage the creation of fresh collagen and blood vessels [28]. By offering a scaffold or matrix that supports the attachment and migration of stem cells to the site of tissue damage, PRP may aid in the recruitment and activation of stem cells, which can develop into different cell types and support tissue repair and regeneration. The extracellular matrix and sticky proteins found in platelets in PRP may serve to facilitate the attachment and growth of stem cells [29]. Moreover, it aids in the activation and stimulation of stem cells' differentiation into particular cell types, such as fibroblasts, which play a role in the regeneration and repair of connective tissue. The fibroblasts, which are the cells responsible for collagen synthesis, can proliferate and differentiate when given the growth factors and cytokines included in PRP. The fibroblasts can be stimulated by the growth factors in PRP to create and deposit new collagen fibers once they have already accumulated at the site of damage [30,31].

In a randomized controlled trial (RCT) including 46 patients, all the participants with persistent plantar fasciitis were given the option of receiving a single injection of PRP or saline as a placebo. At baseline, 2 weeks, 6 weeks, and 3 months following the injection, the VAS score was utilized to measure pain. At all time points, the study indicated that the PRP group's VAS score decreases were noticeably greater than those of the placebo group [33]. In a different similar retrospective cohort trial, 40 patients with chronic severe plantar fasciitis received either a single PRP injection or an injection of corticosteroids as treatment. Pain was measured at baseline, 1, 3, and 6 months following the injection using the VAS score. The study discovered that, at all time points, the VAS values in the PRP group were significantly lower than those in the corticosteroid group [34]. Similar outcomes pertaining to a reduction in VAS scores with PRP treatment were obtained in our study.

A clinical method for assessing the seriousness of foot and ankle issues is the AOFAS score. It can be used to gauge the extent of plantar fasciitis and track how well the treatment is working. The AOFAS score has been utilized in research studies to assess the efficacy of PRP therapy in the treatment of plantar fasciitis. In an RCT, the clinical effectiveness of PRP was assessed in 20 individuals with persistent plantar fasciitis [35]. The patients underwent a single PRP injection and underwent a six-month period of observation. After three and six months after the injection, the researchers saw a significant improvement in AOFAS scores with no severe side events noted. Our study likewise obtained comparable outcomes [36].

The effectiveness of PRP therapy in enhancing the FAAM score, a validated assessment used to assess functional outcomes in patients with foot and ankle disorders, has been the subject of a lot of research [37]. PRP therapy, a placebo, or additional treatments were tested in RCTs on 558 patients with plantar fasciitis. When compared to a placebo or other treatments, PRP therapy significantly increased the FAAM score at the 3, 6, and 12-month follow-ups. Consistent results were found in our study as well, indicating PRP as an effective treatment option for chronic plantar fasciitis [38].

The manner of injection, the volume of blood obtained, and the preparation method all play a role in how well PRP therapy works [39]. Compared to the single injection approach, the peppering technique involves inserting the needle into the target tissue and slowly withdrawing it while keeping the needle tip inside the tissue. The outcome of therapy is improved using this strategy, which is consistent with our study, where the needle is angulated and reinserted to make another puncture onto the fascia at various spots [40].

The relatively small sample size of 25 patients may limit the findings' generalizability. The absence of a control group and the short six-month follow-up period raise questions about causality and long-term outcomes. The heterogeneity in patient characteristics, reliance on subjective measures, selection bias, and the absence of blinding, all introduce potential sources of bias. While the study provides valuable insights, these limitations warrant consideration in interpreting the results and planning future research. The findings of this study on PRP therapy for plantar fasciitis carry substantial implications for clinical practice and research. The demonstrated efficacy of PRP in alleviating pain and enhancing functional outcomes presents a promising alternative for individuals with chronic heel pain unresponsive to conventional treatments. Clinicians can now consider PRP as a valuable intervention, potentially reducing the need for invasive surgical procedures and providing a personalized approach based on individual activity levels and treatment histories. Moreover, the study prompts a reevaluation of treatment protocols, emphasizing the importance of tailoring interventions to patients' unique profiles. Beyond immediate clinical applications, these results guide future research avenues, encouraging investigations into larger cohorts and longer-term effects, paving the way for advancements in musculoskeletal medicine. The broader implications extend to improved patient care, cost-effectiveness considerations, and the ongoing evolution of non-invasive therapies in orthopedics.

## Conclusions

Foot symptoms stemming from plantar fascia deterioration sometimes have PF as their root cause. A better alternative to cautious management should be considered that encourages tissue regeneration. When PRP was utilized to treat PF, every study that was picked and reviewed showed a significant improvement without any signs of problems or adverse effects. The results demonstrate that PRP could be an effective way of treatment for PF. It encourages the creation of new cells and should therefore be considered an effective modality in the treatment of a degenerative condition. PRP was more effective at relieving pain over the long term (24 weeks). Research has demonstrated PRP's superiority over steroid injection over a longer duration of treatment. However, one of the drawbacks of this research is the typically small sample numbers. Moreover, the absence of a placebo, a PF diagnosis, and the length of follow-up may appear to be other factors that limit the ability to evaluate the effectiveness of PRP.
